# Automation of classification of sleep stages and estimation of sleep efficiency using actigraphy

**DOI:** 10.3389/fpubh.2022.1092222

**Published:** 2023-01-09

**Authors:** Hyejin Kim, Dongsin Kim, Junhyoung Oh

**Affiliations:** ^1^College of Pharmacy, Sookmyung Women's University, Seoul, Republic of Korea; ^2^NYX Inc., Gyeonggi-do, Republic of Korea; ^3^Center for Information Security Technologies, International Center for Conversing Technology Building, Anam Campus (Science), Korea University, Seoul, Republic of Korea

**Keywords:** sleep scoring, sleep efficiency, actigraphy, machine learning, algorithm

## Abstract

**Introduction:**

Sleep is a fundamental and essential physiological process for recovering physiological function. Sleep disturbance or deprivation has been known to be a causative factor of various physiological and psychological disorders. Therefore, sleep evaluation is vital for diagnosing or monitoring those disorders. Although PSG (polysomnography) has been the gold standard for assessing sleep quality and classifying sleep stages, PSG has various limitations for common uses. In substitution for PSG, there has been vigorous research using actigraphy.

**Methods:**

For classifying sleep stages automatically, we propose machine learning models with HRV (heart rate variability)-related features and acceleration features, which were processed from the actigraphy (Maxim band) data. Those classification results were transformed into a binary classification for estimating sleep efficiency. With 30 subjects, we conducted PSG, and they slept overnight with wrist-type actigraphy. We assessed the performance of four proposed machine learning models.

**Results:**

With HRV-related and raw features of actigraphy, Cohen's kappa was 0.974 (p < 0.001) for classifying sleep stages into five stages: wake (W), REM (Rapid Eye Movement) (R), Sleep N1 (Non-Rapid Eye Movement Stage 1, S1), Sleep N2 (Non-Rapid Eye Movement Stage 2, S2), Sleep N3 (Non-Rapid Eye Movement Stage 3, S3). In addition, our machine learning model for the estimation of sleep efficiency showed an accuracy of 0.86.

**Discussion:**

Our model demonstrated that automated sleep classification results could perfectly match the PSG results. Since models with acceleration features showed modest performance in differentiating some sleep stages, further research on acceleration features must be done. In addition, the sleep efficiency model demonstrated modest results. However, an investigation into the effects of HRV-derived and acceleration features is required.

## 1. Introduction

Good sleep habits impact everyday lives, including daytime performance, mood, confidence, and relationships with others. In hospitals, sleep analysis is essential for identifying problems related to sleep-wake disorders. It is also interrelated with physiological analysis, as well as with psychological one. For finding out sleep problems, sleep stage identification with the help of PSG (polysomnography) is required (1). Classification of sleep stages is the process of sorting sleep into several stages. Since scoring of the sleep stage has been used for the diagnosis of several sleep disorders (2), it has been the gold standard for analyzing sleep clinically. There have been various methods for PSG recording, for instance, EEG (electroencephalogram), EMG (electromyogram), and EOG (electrooculogram). Even though PSG provides a wide range of data about sleep, including sleep stages, it is prone to be regarded as obtrusive or sometimes invasive. In addition, classifying sleep stages is an arduous task since persistent observation is required to collect and analyze clinical data.

For sleep stage classification, the EEG has been frequently used in both manual scoring and automated classification (3, 4). Although using multiple EEG channels could increase the accuracy of sleep scoring, it can be uncomfortable to wear many electrodes during sleep. Research using devices with a single EEG channel would solve that problem (4, 5). Many research using EEG has utilized conventional methods of machine learning. However, recently, deep learning techniques are applied to perform the classification tasks (6). For example, convolutional neural networks (CNNs) or Recurrent Neural Networks (RNNs) were designed and utilized (7).

Heart rate variability (HRV) is one of the alternatives for PSG and is a parameter of the autonomic nervous system, which could be obtained by measuring electrocardiography (ECG) (8). There have been various studies about the deduction of sleep stages using machine learning algorithms, which convert HRV features into sleep stages. Most of them concentrated on sleep/wake or wake/REM (Rapid eye movement)/NREM (non-REM) classification (9–11). There are two critical components for automatic sleep stage classification: feature extraction and machine learning algorithm. Techniques for feature extraction could vary by type of recording; for instance, frequency-domain analysis and time-frequency-domain analysis have been used in EEG analysis (12). Using EEG, EMG, or EOG signals might be better in terms of performance, but it is less convenient for in-home sleep studies. They require more special equipment settings for data acquisition. In addition, EEG electrodes are especially difficult to set up by themselves. A viable alternative for those methods could be the ECG. There has been researching on an algorithm for detecting QRS and measuring HRV in ECG (13). ECG-related techniques for sleep scoring called the progressive detrended fluctuation analysis (PDFA) were introduced by Tesler et al. (14, 15). The PDFA was based on the DFA (detrended fluctuation analysis) method and could catch the transition sensitively, but it needed to propose an accurate scheme for sleep staging. In one study conducted with single-lead ECG, windowed detrended fluctuation analysis (WDFA) was used for sleep scoring and estimating sleep efficiency. They utilized RR series, which were derived from ECG data, for feature extraction, and features were selected based on the SVM (Support vector machine) recursive features elimination method (16).

Photoplethysmography (PPG)-based methods were used in several studies for distinguishing wake, sleep, or REM sleep (17, 18). The HRV could be derived from PPG sensors.

Actigraphy, which comprises a storage unit and an accelerometer, was proposed as one of the surrogate modalities for PSG (19). Actigraphy has become an effective tool for assessment in sleep research for decades, owing to its usefulness. Its application includes the diagnosis and treatment of specific physiological and neurological disorders. Actigraphy can also be applied to assess the efficacy of pharmacologic and non-pharmacologic therapies (20). Human sleep consists of distinct stages like REM sleep or NREM sleep stages. It has been known that people feel less fatigue when they wake up during the REM period. Continuous sleep monitoring, therefore, would be possible if sleep patterns are analyzed based on body state and environmental information with an artificial intelligence system and if an optimized pattern model is developed by applying extrinsic environmental factors and sleep stages. When the ECG signals were used to support sleep stage classification, the accuracies varied from 56 to 89%. Studies with EEG signals generally showed higher accuracies, which varied from 81 to 98%. However, the studies with the highest accuracies selected two or three-class problems (21). In one study conducted with patients with suspected sleep apnea, the classification model with PPG signals showed 64.1% accuracy (kappa = 0.51) (22).

There has been vibrant research about systems for monitoring or quantifying sleep quality. One automatic monitoring system proposed by Zhu et al. (23) utilized a piezoelectric transducer for sensing the user's cardiac impulse, respiration, and physical movements. The sensor was placed under a mattress, and data collected from sensors was sent to database servers and processed. The noninvasive model for quantifying sleep quality utilized an accelerometer and a sensor for pressure. They selected several parameters, including heart rate, body movement, and respiration, for assessing sleep quality (24). Previous research with actigraphy devices (Fitbit) has shown controversial sleep efficiency estimation results (25). Several studies argued that the estimated sleep efficiency was overestimated compared to the PSG or EEG-based method (26, 27). However, there were contrary arguments (28, 29). Although few studies have quantified the accuracies, actigraphy devices can estimate sleep efficiency with an accuracy of 86% (30).

This research aims to prevent early awakening and estimate optimal wake-up time by improving the precision of sleep pattern analysis. Estimating sleep stages would be faster and more economical than PSG as long as the process is automatized with artificial intelligence learned from extensive data, for instance, Photoplethysmography (PPG) data. This research aims at developing a multinomial classification machine learning algorithm that can predict sleep stages with PPG and actigraphy. In this study, classifications with PSG data were hypothesized to be true. Through this study, not only do we propose novel models with good performance, but we could investigate the effect of HRV or acceleration-derived features on performances.

## 2. Materials and methods

### 2.1. Sleep stage classification model

**Figure 1:**
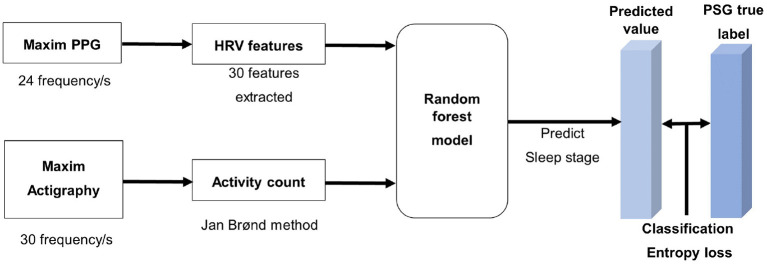
This is a process flow diagram of sleep stage classification model.

Generally, a sleep study is conducted under the Rechtschaffen and Kales (R&K) or a new standard developed by The American academy of sleep medicine (AASM). We followed the AASM standard, which divides sleep into five stages: wake (W), rapid eye movement (REM), and three sleep stages (S1, S2, and S3). The Maxim band is a wrist-worn actigraphic device that utilizes optical components and accelerometers to measure users' health metrics. Since it contains the PPG sensor and related features, it could measure vital signs (e.g., heart rate, respiration rate) more accurately than those without PPG sensors. In addition, it is unobtrusive to wear while sleeping compared to other devices using EEG recordings. The analysis process flow for sleep stage classification is described in Figure 1. Tree-based ensemble model (random forest) (31–33) was utilized for processes that are described below. The measuring or transforming velocity of the Maxim PPG data (24 frequency/s) was unsuitable for the conventional algorithm (30 frequency/s) (34) used in actigraphy research. So, the PPG and actigraphy datasets were created separately.

### 2.2. Sleep efficiency predicting model

**Figure 2:**
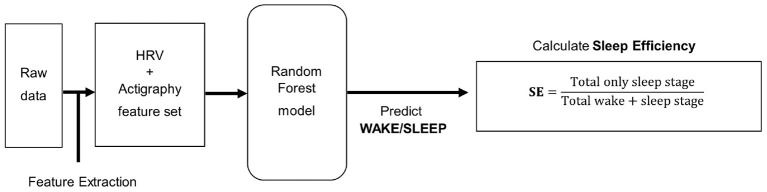
This is a process flow diagram of sleep efficiency predicting model.

The analysis process flow for predicting sleep efficiency is depicted in Figure 2. Like the sleep stages predicting model, the raw data of PSG was merged with the Maxim raw data based on time, and the same feature extraction process was utilized. In addition, the random forest model targeted for binary classification of wake/sleep was applied for predicting the wake and sleep state. The overall analysis pipeline utilizes this result for obtaining sleep efficiency.

### 2.3. Data collection and dataset creation

**Figure 3:**
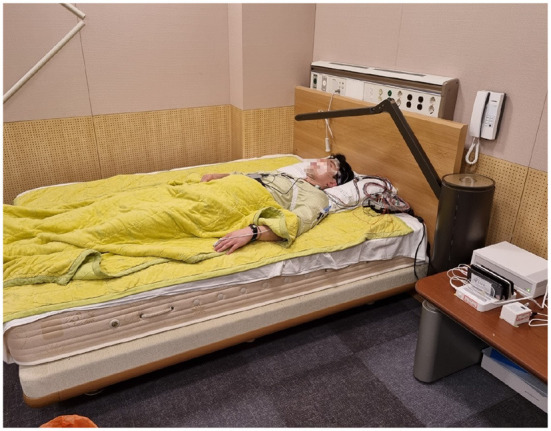
This is a picture of the environment for the test. One subject is wearing actigraphy devices and electrodes for PSG.

All study procedures were approved by the Samsung Medical Center Institutional Review Board (Seoul, Republic of Korea) in respect of ethics and science. The eligibility criteria for inclusion were 20–65 years old adults who have trouble with sleep onset, fully understand the objective of this research, and Android users or iPhone users who could use Wi-Fi in their bedrooms. Those who had current psychiatric or neurologic disorders, cognitive impairment, pulmonary diseases including obstructive lung disease, severe medical illnesses that could not be clinically controlled (heart, kidney, nerve, gastrointestinal tract, diabetes, hypertension, thyroid, immunodeficiency, and cancer), severe snoring, narcolepsy, and REM sleep disorder were excluded. Pregnant or lactating women, shift workers, and those already diagnosed with insomnia were also excluded from this study. The clinical trial was conducted with 30 people. Since the Maxim band measures 24 times per second, and the average measurement duration was 8 h, 20,736,000 recordings were generated from 30 subjects. Hence, it was enough for the artificial intelligence model to learn. All subjects wore Maxim bands for collecting PPG data, and simultaneously, PSG was conducted in the Sleep Center in Samsung Medical Center (Figure 3). During their PSG evaluation, electrodes were attached to subjects. PPG and PSG data were automatically saved and processed through software for sleep pattern and efficiency prediction.

A merge (integration of data based on the column) was conducted in creating datasets. In interpreting PSG data, each epoch of 30 s is classified into several sleep stages. Because the Maxim band measures 24 times per second, two sets of data had to be merged based on the PSG data for synchronization. First, these 30-s dataset units based on time columns were tested for estimating the value of PSG sleep stages as “true.” Second, real-time PSG data of 30 subjects were processed into a single dataset based on each row.

### 2.4. Pre-processing of accelerometry and ECG data

#### 2.4.1. Acceleration features

The x-, y-, and z-axis data among whole Maxim data were utilized for extracting activity count. Compared with the Maxim band, which measures 24 times per second, the traditional Jan Brønd algorithm (35) measures 30 times per second. Therefore, the raw data was resampled and processed according to the Jan Brønd algorithm.

#### 2.4.2. HRV-related features (ECG features)

RR columns (RR peak intervals in the QRS wave of ECG) of the Maxim raw data were utilized for extracting HRV-related features. RR peak values excluding zero were extracted for use in HRV feature extraction.


RR peak values≤700

were considered outliers and excluded, and after the data was interpolated using the linear method, ectopic beats were deleted with Malik's method (36). In RR peak data, if the window size was set up as 10, the moving average trend was in an acceptable range. As a result, we concluded that the trend of the data was reflected in that window size, continuing the HRV feature extraction.

### 2.5. Feature extraction

From raw data characteristic variables, derived variables were created for the recognition from various angles of the machine learning model. In this process, HRV-related and actigraphy-related derived variables were formed.

#### 2.5.1. Acceleration features

The function was designed for extracting activity counts based on the x-, y-, and z-axis data. In the process, signals were filtered using a predefined filter coefficient, which Jan Brønd used (34, 35).

#### 2.5.2. HRV-related features (ECG features)

About 29 HRV-derived variables were created in several domains: time domain, frequency domain, geometrical domain, and non-linear domain. Among those variables, those with high importance were listed in Table 1. FFT Spectrum (Welch's periodogram) based on these derived variables and Lorentz Plot were obtained.

**Table 1:** Important variables among HRV variables.

**Domain**	**Feature name**	**Expression**
Time	Mean/Std/Max HR	Average of heart rate/standard deviation/maximum
	SDNN	Standard deviation of the normal-to normal interval
	NN50	Number of NNi differences 50 ms
	Range NNi	Gap between maximum and minimum RR
	CVSD	Dispersion coefficient of successive difference
Geometric	Triangular index	Integral value of the density distribution
Frequency	Low/high frequency	Variance in HRV in the low/high frequency
	Mean NNi	Mean of RR intervals
	VLF	Variance in low frequency
	LF/HF	Low/high frequency ratio
Non-linear	SD1, SD2	Standard deviation of the Poincare plot
	Cardiac vagal IndeNx	Cardiac vagal IndeNx

### 2.6. Classification of sleep stages

#### 2.6.1. Algorithm selection

The model selection was implemented based on derived variables acquired after pre-processing for selecting an optimized machine learning algorithm. After carrying out an analysis and comparison among 13 machine learning algorithms, including simple linear algorithms (Linear Discriminant Analysis, Logistic Regression) and tree-based models (Decision tree, LightGBM, and random forest), the algorithm with the best performance was determined as AutoML, which is based on reinforcement learning.

#### 2.6.2. Modeling

There are discrepancies in measurement units between the HRV dataset and actigraphy data because of the resampling process. Thus, each dataset went through additional modeling, divided into two cases for assessing the influence of the generated derived variables: Including both the derived variables and the Maxim raw data feature and including only derived variables. After several experiments and the process of AutoML model selection, the machine learning model was decided as a random forest, a tree-based ensemble model. The result of the model evaluation was diagnosed with a confusion matrix, and verification of feature effectiveness was conducted with the feature importance following random forest entropy.

### 2.7. Sleep efficiency prediction model

#### 2.7.1. Dataset creation

Since the objective of this analysis is the binary classification of each sleep stage, among five sleep stages from PSG data, all four stages except “wake” were relabeled as “sleep.” Thus, the dataset relabeling wake/sleep as 0/1 was conducted, reflecting the target of the machine learning model. Furthermore, each dataset for every subject was created after this relabeling, and the prediction accuracy was estimated individually. Feature extraction was conducted similarly to the sleep stages predicting analysis. HRV-derived variables and actigraphy-derived variables were produced separately. Among these, significant variables based on the feature importance were selected and applied for the modeling.

#### 2.7.2. Machine learning modeling

The machine learning modeling was implemented based on the pre-processed dataset and derived variables. The same random forest model was also used in this research because it showed an optimal performance by AutoML in predicting sleep stages. Whereas the sleep stage predicting model adopted the multiclass classification, this model focused on the binary classification of sleep stages into wake and sleep. Each data of 30 subjects went through binary classification, and the subsequential accuracy of the classification was estimated individually.

#### 2.7.3. Calculation for sleep efficiency

PSG data (PSG was conducted in a hospital setting, and the PSG data is split into 30-s epochs) of subjects were considered the standard sleep efficiency (standard SE). Our sleep efficiency prediction software was implemented with subjects' data: subjects' sleep data were input, each section was checked, and predictions for sleep efficiency proceeded. Sleep efficiency (SE) and the accuracy in predicting sleep efficiency were calculated by the equation as follows.


(1) $$\begin{array}{l} Sleep\;efficiency\;\left(\%\right)=\frac{Total\;sleep\;time\;\left(s\right)}{total\;minutes\;in\;bed\;\left(s\right)} \end{array}$$

(2) $$\begin{array}{l} Accuracy\;in\;predicting\;sleep\;efficiency\;\left(\%\right)\\ \;=\left(1-\frac{Standard\;SE\;\left(\%\right)-Estimated\;SE\;\left(\%\right)}{Standard\;SE\;\left(\%\right)}\right)\times 100 \end{array}$$

### 2.8. Statistical analysis

Statistical analyses were performed with SPSS version 26.0 (SPSS, Inc., Chicago, IL, USA). Cohen's Kappa coefficient was used for the sleep stage classification model to assess classification accuracy. Furthermore, the average and SD (standard deviation) of standard and estimated sleep efficiency were analyzed. In addition, paired *t*-test was conducted to assess the performance of our model. The results were statistically significant for *p* < 0.05.

## 3. Results

### 3.1. Participants

The demographic information of 30 subjects is shown in Table 2. The mean age of participants was 44.1 (26–62 years old). After being recruited, clinicians diagnosed whether or not they had sleep disorders based on the PSG results.

**Table 2:** Demographic information of 30 subjects.

**Sex**	**Subjects**	**Age**	**Subjects**		**Median (IQR)**
Male	17 (57%)	21–30	2	Age (years)	26 (18.75)
		31–40	13	Sex (M/F)	17/13 (57% M)
		41–50	5	Diagnosis of sleep disorders	8/12 (40%)
Female	13 (43%)	51–60	8		
		61–70	2		
Total				30	

### 3.2. Sleep stage classification

#### 3.2.1. Algorithm selection

The performance of machine learning algorithm models measured based on accuracy, recall, and F1 score is depicted in Table 3. After model selection with AutoML, all tree-based models (DT, RF, and LightGBM) were superior to simple linear algorithms (SVM and LDA), resulting in an accuracy of about 0.90. Therefore, this analysis selected the random forest ensemble machine learning, which was remarkable in terms of AUC, Recall, and F1 scores among various models.

**Table 3:** The performance of machine learning algorithm models.

	**Model**	**Accuracy**	**AUC**	**Recall**	**Prec**.	**F1**	**Kappa**	**MCC**	**TT (s)**
et	Extra Trees Classifier	0.9939	0.9999	0.9849	0.9939	0.9938	0.9911	0.9911	1.146
rf	Random Forest Classifier	0.9890	0.9998	0.9739	0.9891	0.9889	0.9839	0.9840	1.868
lightgbm	Light Gradient Boosting Machine	0.9932	0.9998	0.9843	0.9933	0.9932	0.9901	0.9902	1.258
gbc	Gradient Boosting Classifier	0.9100	0.9833	0.8410	0.9119	0.9082	0.8666	0.8681	14.457
knn	K Neighbors Classifier	0.9049	0.9788	0.8605	0.9049	0.9045	0.8611	0.8613	0.187
dt	Decision Tree Classifier	0.9688	0.9773	0.9513	0.9691	0.9688	0.9546	0.9547	0.120
lda	Linear Discriminant Analysis	0.6225	0.7614	0.4109	0.6289	0.5886	0.4015	0.4177	0.086
nb	Naïve Bayes	0.4597	0.7056	0.3780	0.5173	0.4302	0.2609	0.2901	0.041
ada	Ada Boost Classifier	0.5325	0.6531	0.3914	0.5395	0.5286	0.3144	0.3180	0.808
lr	Logistic Regression	0.4729	0.6490	0.2560	0.3235	0.3676	0.1063	0.1347	5.781
ridge	Ridge Classifier	0.6198	0.0000	0.3810	0.6357	0.5714	0.3860	0.4087	0.038

#### 3.2.2. Machine learning modeling

**Figure 4:**
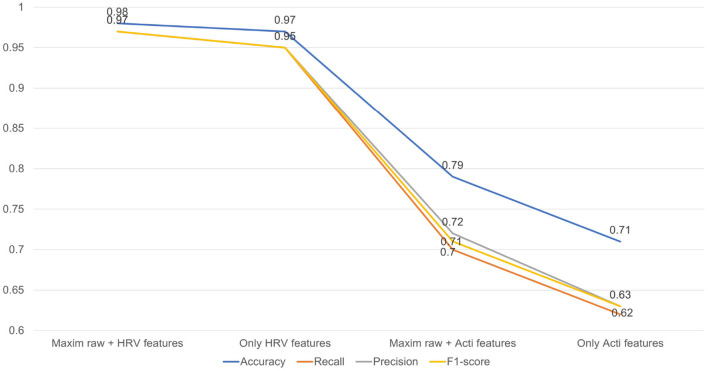
Performance of four machine learning modelings were depicted as a line graph.

The overall performance of four kinds of modeling is summarized in Figure 4.

##### 3.2.2.1. Acceleration features

First, the activity count-derived variables and the Maxim raw data features were included for modeling. The confusion matrix of this model is given in Table 4. The kappa coefficient was 0.682 (*p* <0.001), meaning there was substantial agreement between predicted and true labels. A valid accuracy of 0.79 was acquired by modeling with an activity count-included dataset based on the x-, y-, and z-axis. Other indices related to performance were 0.70 (Recall), 0.72 (Precision), and 0.71 (F1-score). However, the result of the confusion matrix showed that sleep stage N1 (S1) and sleep stage N2 (S2) were not sorted clearly. Second, only the activity count-derived variables were included. The modeling with a dataset consisting of only the actigraphy-related variables was implemented to assess the influence of those variables. The confusion matrix of this modeling is depicted in Table 5. As a result, the kappa coefficient was 0.568 (*p* <0.001), meaning there was moderate agreement. The valid accuracy was also decreased to 0.71; Other performance factors were 0.62 (Recall) and 0.63 (Precision and F1-score).

**Table 4:** Confusion matrix of the model that includes the Maxim raw features and the activity count-derived variables.

True label
	REM	109	0	1	8	0
	Wake	1	34	8	9	0
	S1	2	13	16	32	1
	S2	9	6	7	251	13
	S3	1	0	2	10	46
		REM	wake	S1	S2	S3
		Predicted label				

**Table 5:** Confusion matrix of one model that includes only the activity count-derived variables.

True label
	REM	97	3	3	15	0
	Wake	6	29	5	12	0
	S1	2	12	16	33	1
	S2	15	10	15	228	18
	S3	0	1	1	16	41
		REM	wake	S1	S2	S3
		Predicted label				

##### 3.2.2.2. HRV-related features (ECG features)

When both the Maxim raw data features and the HRV-derived variables were included, the model showed good performance with a kappa coefficient of 0.974 (*p* <0.001), showing almost perfect agreement. The confusion matrix of this model is shown in Table 6. In addition, the valid accuracy was 0.98, and the recall, precision, and F1-score were all 0.97. Especially raw features, including Green count and IR count, showed outstanding performances, and among the derived variables, the performance of median_nni and min_hr was outstanding. Green count, which had the most significant importance, showed significantly different values in each sleep stage. Second, only the HRV-derived variables were included. For evaluating the influence of HRV-derived variables, modeling was implemented with a train/valid dataset that consists of only derived variables, not the Maxim raw data features. The model showed almost perfect agreement, with the kappa coefficient of 0.951 (*p* <0.001). The confusion matrix of this model is presented in Table 7. The valid accuracy was 0.97, implying still excellent accuracy, although the figure was lower than that of the other case, including the Maxim feature. Among numerous variables, min_hr and median_nni were the most important for classifying sleep stages. In addition, recall, precision, and F1-score were obtained as 0.95.

**Table 6:** Confusion matrix of the model that includes the Maxim raw features and the HRV-derived variables.

True label
	REM	445	1	0	2	0
	Wake	3	100	2	1	0
	S1	1	6	136	4	0
	S2	4	2	4	816	0
	S3	0	0	0	0	184
		REM	wake	S1	S2	S3
		Predicted label				

**Table 7:** Confusion matrix of one model that includes only the HRV-derived variables.

True label
	REM	433	5	1	9	0
	Wake	7	94	3	2	0
	S1	5	7	131	4	0
	S2	8	3	2	813	0
	S3	0	0	0	0	184
		REM	wake	S1	S2	S3
		Predicted label				

### 3.3. Sleep efficiency prediction

#### 3.3.1. Machine learning modeling

The accuracy of the estimated sleep efficiency of 30 subjects was calculated. The final average accuracy was about 86.19%, implying that the estimated predicting efficiency of sleep also shows an accuracy of about 86.19%.

#### 3.3.2. Calculation for sleep efficiency

The mean standard SE, calculated using the PSG data of 30 subjects, was 85.11 ± 6.48% and the mean estimated SE using the proposed modeling was 73.41 ± 8.18%. The accuracy in predicting sleep efficiency (%) could be calculated by Equations (1) and (2). The mean accuracy for estimating sleep efficiency was 86.19 ± 6.07 %. The standard SE and estimated SE were compared by paired *t*-test. The estimated SE was significantly lower than the standard SE (*p* <0.001), and the difference between them was 11.70%.

## 4. Discussion

The tree-based random forest algorithm, which showed remarkable performance in terms of AUC, Recall, and F1-score, as well as accuracy, was chosen in this study. We made four classification models utilizing the random forest method. Overall, both models using the HRV-related features performed better than the others. Sleep stage classification results were almost perfectly matched with the PSG results (kappa = 0.974, 0.951). Also, they perfectly distinguished the sleep N3 (S3) stage. The model with the Maxim raw features and HRV features was the best in terms of kappa, accuracy, recall, precision, and F1-score. However, all performance indices were decreased when the Maxim raw features were excluded. Especially the precision and recall for distinguishing the wake (W) stage were significantly influenced. Comparing these two models shows that the Maxim raw features are involved in distinguishing between sleep and wake stages. Nevertheless, the model with only HRV-related features showed good performance, still.

On the other hand, models with acceleration features showed poorer performance than the former ones. They showed substantial or moderate agreements between true and estimated labels. Regardless of whether it includes the Maxim raw features, they failed to distinguish between the S1 and S2 stages. When the Maxim raw features were included, the precision and recall for classifying the S1 stage were 0.47 and 0.25. The precision was further decreased to 0.40 when the Maxim raw features were excluded. As sleep progresses to deeper stages, activity decreases, so it seems that sleep stages are difficult to be distinguished based on the activity. The result that the REM stage was relatively well-distinguished by these models supports that interpretation. Nevertheless, models using HRV features successfully differentiate between S1 and S2, and their performances were significantly better than those of the acceleration models. So, Further studies about these acceleration features must be done to overcome these limitations.

The mean accuracy for estimating sleep efficiency was 86.19 ± 6.07%. Compared with other research conducted with OSA (obstructive sleep apnea) patients, the level of agreement between the standard SE and estimated SE was not better in our research. The OSA research used a WP100 device containing a peripheral arterial tonometer and an oxygen saturation sensor, as well as ASWA (sleep/wakefulness analysis software) (37). Because that research used more sensors than our research, the accuracy could differ. However, it could be interpreted that a more accurate estimation would be achievable with superior sensors in the near future. In addition, the SE estimation model contains both HRV-derived and activity count-derived features, and the acceleration features showed only moderate performances on sleep stage classification. As a result, it could decrease the accuracy of binary classification. Therefore, more research must be conducted to investigate the effect of HRV-derived and acceleration features on the sleep stage scoring processes. Lastly, the sample size of 30 could not be enough to assess the accuracy. In addition, the subjects were a heterogenous population because some participants were diagnosed with sleep disorders during the clinical trial. A larger scale of the test might be needed for more accurate estimation results.

Also, as the number of wakefulness increases while sleeping, the correlation in PSG-actigraphy tends to be weakened (38, 39). Hence, for those with fragmented sleep patterns, this poor capability of identifying wakefulness can be an obstacle in using actigraphy (20). Most of our participants (93%) showed the number of awakenings over 4 (which is not described in the results), demonstrating they might not sleep well in the strange sleep environment. Based on these, the hospital environment could contribute to the low accuracy of classifying the awake state. Since subjects had to sleep overnight for PSG and actigraphy data recording, they might have more fragmented sleep than usual. Therefore, it could explain the low accuracy of the awake stage and the discrepancy of PSG-actigraphy in sleep efficiency.

Our research demonstrated the possibility of automation of sleep stage classification and estimation of sleep efficiency using actigraphy devices. Although classification models with acceleration features showed moderate performance in distinguishing the S1 and S2 stages, models with HRV-related features classified each stage precisely, resulting in almost perfect matches. Also, sleep research with machine learning algorithms and deep learning has been conducted vibrantly (40). Not only does this research help healthy people, it could also help patients with various problems (41). Even though the PSG is the gold standard so far, the use of actigraphy has been increasing in clinical settings. Mainly the unobtrusive actigraphy devices have been frequently used for severely ill patients (42). Sleep deprivation in these ICU (intensive care unit) patients is associated with adverse outcomes, so monitoring sleep quality by actigraphy has importance in terms of patients' status. However, among numerous studies with actigraphy, there was no consistency in device type, epoch length, related software, and measuring parameters among sleep research with actigraphy devices. Thus, it is not easy to compare the results of those studies, including this research. Studies using actigraphy devices, therefore, need common standards for fostering sleep research. Furthermore, if these actigraphy-based methods work with IoT devices, a ubiquitous system for managing sleep could be realized shortly. Recent studies are working on designing and constructing sleep monitoring systems (43, 44). Careful sleep monitoring with devices could prevent certain types of sleep disorders, saving a large portion of healthcare costs.

## Data availability statement

The raw data supporting the conclusions of this article will be made available by the authors, without undue reservation.

## Ethics statement

The studies involving human participants were reviewed and approved by Samsung Medical Center. The patients/participants provided their written informed consent to participate in this study.

## Author contributions

HK and JO contributed to conception and design of the study. HK performed the formal analysis, data curation, and wrote the first draft of the manuscript. JO supervised the overall research and administrated the whole project. DK acquired the funding and resources. All authors contributed to manuscript revision, read, and approved the submitted version.
